# Preterm Prelabour Rupture of Membranes before Viability in Twin Pregnancies: What Can We Expect?

**DOI:** 10.3390/jcm12082949

**Published:** 2023-04-18

**Authors:** Júlia Ponce, Teresa Cobo, Clara Murillo, Anna Gonce, Nadia Domínguez, Francesca Crovetto, Laura Guirado, Montse Palacio, Mar Bennasar

**Affiliations:** 1 BCNatal, Fetal Medicine Research Center, Hospital Clínic and Hospital Sant Joan de Déu, 08028 Barcelona, Spain; ponce@clinic.cat (J.P.); tcobo@clinic.cat (T.C.); cmurillo@clinic.cat (C.M.); agonce@clinic.cat (A.G.); nadominguez@clinic.cat (N.D.); bennasar@clinic.cat (M.B.); 2 Institut de Recerca Sant Joan de Déu, 08950 Esplugues de Llobregat, Spain

**Keywords:** multiple pregnancy, preterm birth, preterm prelabour rupture of membranes, periviability, chorionicity, latency, perinatal mortality

## Abstract

Preterm prelabour rupture of membranes (PPROMs) before viability carries significant perinatal mortality and morbidity. Clinical management and prenatal counselling are a challenge, especially in twin pregnancies, due to scarce evidence on how previable PPROM affects this population. The aim of this study was to describe pregnancy outcomes of twin pregnancies complicated with previable PPROM and evaluate potential prognostic factors that may predict perinatal mortality. A retrospective cohort including dichorionic and monochorionic diamniotic twin pregnancies complicated with PPROM before 24 + 0 weeks of pregnancy was evaluated. Perinatal outcomes of pregnancies managed expectantly were described. Factors predicting perinatal mortality or reaching periviability (defined from 23 + 0 weeks onwards) were evaluated. Of the 45 patients included, 7 (15.6%) spontaneously delivered within the first 24 h after diagnosis. Two patients (5.3%) requested selective termination of the affected twin. In the 36 ongoing pregnancies that opted for expectant management, the overall survival rate was 35/72 (48.6%). There were 25/36 (69.4%) patients who delivered after 23 + 0 weeks of pregnancy. When periviability was achieved, neonatal survival increased up to 35/44 (79.5%). Gestational age at delivery was the only independent risk factor of perinatal mortality. The overall survival rate of twin pregnancies complicated with previable PPROM is poor but similar to singletons. No prognostic factors, apart from achieving periviability, were identified as individual predictors of perinatal mortality.

## 1. Introduction

Preterm prelabour rupture of membranes (PPROMs) accounts for 25–30% of preterm births [1]. Multiple pregnancy is a relevant contributor to both conditions. On one hand, PPROM affects 7–11% of twin pregnancies compared to 2–4% of singletons. On the other hand, twins account for 10–15% of global prematurity [2,3,4].

The presentation of PPROM before 24 + 0 weeks of pregnancy is rare and affects less than 0.5% of all pregnancies, but it carries significant perinatal mortality and morbidity, and the impact is further exacerbated when there is a multiple pregnancy [3,4]. Prenatal management and counselling of previable PPROM in twins are a challenge for maternal–foetal medicine specialists due to scarce evidence on how previable PPROM affects this population [5]. Indeed, most studies are based on singleton series, with few cases of multiple pregnancies. Furthermore, to date, no study has assessed predictive factors of perinatal mortality in twin pregnancies complicated with previable PPROM.

Regarding singleton population, different series report a wide neonatal survival rate (32–63%) [6]. In addition to the complications of extreme prematurity, there is the potential risk of pulmonary hypoplasia and early-onset sepsis due to long-standing PPROM [6,7,8,9]. Moreover, PPROM increases the risk of emergency delivery due to an increased risk of clinical chorioamnionitis, placental abruption and cord prolapse [7], which could explain the increased maternal morbidity. Gestational age at delivery and severe and prolonged oligohydramnios are considered relevant determinants for predicting perinatal mortality in previable PPROM [6,10]. However, there is little evidence of the impact of these factors on perinatal mortality and morbidity in cases of previable PPROM in multiple pregnancies. Therefore, prenatal counselling and clinical management of PPROM in twins is controversial and is conditioned by the fact that there is another healthy foetus that can be affected by prematurity or infectious complications of PPROM. To date, these patients are counselled with expectant management under antibiotic treatment or the selective reduction in or termination of the entire pregnancy [11,12].

The aim of this study was to describe pregnancy outcomes of twin pregnancies complicated with previable PPROM and evaluate potential prognostic factors that may predict perinatal mortality in order to improve the prenatal counselling of these pregnancies.

## 2. Materials and Methods

### 2.1. Study Population

This was a retrospective study including dichorionic and monochorionic diamniotic twin pregnancies complicated with spontaneous or procedure-related PPROM before 24 + 0 weeks of pregnancy, admitted to our centre between 2015 and 2022. Data collection was included under the study HCB/2020/0192 approved by the Ethics Committee of our hospital. The exclusion criteria were delivery <24 h within PPROM diagnosis, intrauterine foetal demise (IUFD) of one foetus before PPROM diagnosis, clinical chorioamnionitis defined according to Gibbs criteria at PPROM presentation [13], monoamniotic pregnancies, triplets, or higher order pregnancies. Cases with major structural or chromosomal anomalies, elective termination of pregnancy for maternal or foetal reasons not related to PPROM were also excluded. With regard to monochorionic pregnancies that underwent a foetoscopy, cases with transient amniotic fluid leakage were not included in this study.

### 2.2. Outcomes

Primary outcomes were gestational age at delivery, latency (weeks) from PPROM to delivery, and overall perinatal survival rate (miscarriage (considered as delivery <23 + 0 weeks), IUFD, and neonatal death). Composite neonatal morbidity included bronchopulmonary dysplasia, early-onset sepsis, grade III–IV intraventricular haemorrhage, necrotizing enterocolitis, and neonatal death. Long-term neonatal complications were not included, as they were not feasible to report adequately due to the external follow-up of most children. Foetal and maternal complications related to PPROM, such as pulmonary hypoplasia, clinical chorioamnionitis, cord prolapse, placental abruption, severe maternal postpartum haemorrhage, severe infection, maternal intensive care unit admission, or hysterectomy, were recorded as secondary outcomes. Perinatal mortality was defined as foetal or neonatal death between 20 + 0 weeks and the first 28 days of life, whereas periviability was considered from 23 + 0 weeks onwards.

### 2.3. Data Collection

Data were collected from patients’ medical files. Baseline characteristics included chorionicity, maternal age, ethnicity, body mass index, use of assisted reproductive technology (ART), nulliparity, and risk factors for preterm birth such as smoking, previous spontaneous preterm birth, uterine malformation, and cone biopsy. A history of previous invasive procedures (chorionic villus sampling, amniocentesis, cervical cerclage, or foetoscopic surgery for twin-to-twin transfusion syndrome) was also recorded. Gestational age was based on the crown-rump length of the larger twin at the 11–14-week scan. Chorionicity was determined based on the presence of the “T” or “lambda” sign before 14 weeks.

### 2.4. PPROM Assessment and Management

The diagnosis of PPROM was based on visualization of amniotic fluid leaking through the external cervical os on speculum examination and confirmed by vaginal pH or detection of insulin-like growth factor-binding protein-1 using an immunochromatographic test (Amnioquick, Biosynex SA, Illkirch-Graffenstaden, France).

Maternal blood tests, including white blood cell (WBC) count and C-reactive protein (CRP) levels, and urine culture were routinely performed in all patients at admission. Cervical and vaginal cultures were only performed if cervical cerclage and/or anomalous vaginal discharge were present at physical examination. Cervical length and maximum vertical pocket of amniotic fluid (MVP-AF) were measured at PPROM diagnosis and monitored weekly by ultrasound assessment in all patients until delivery. Amniocentesis of the PPROM-foetus, to rule out intra-amniotic infection at admission or anytime during pregnancy, was performed according to the clinician’s criteria considering blood test results and clinical findings.

All patients received antibiotics (intravenous ampicillin 1 g/6 h and gentamicin 80 mg/8 h and a single dose of azithromycin 1 g, until 2019, and intravenous ampicillin 2 g/6 h and ceftriaxone 1 g/12 h and oral clarithromycin 500 mg/12 h, from 2020 onwards) at hospital admission for five days. Bed rest was advised during the first 48 h followed by relative rest thereafter. Betamethasone 12 mg/24 h (two doses) was administered for lung maturation after 24 + 0 weeks if delivery was expected in the subsequent 7 days (i.e., presence of uterine contractions, vaginal bleeding, cervical length < 25 mm, and/or blood test alterations suggesting infection). Considering the current controversy related to foetal viability, lung maturation was considered between 23 + 0 and 23 + 6 weeks in selected cases if immediate delivery was expected and parents requested active neonatal resuscitation after extensive neonatal counselling. Tocolytics were administered in cases of preterm labour and no contraindication to prolong pregnancy (i.e., no signs of chorioamnionitis or placental abruption). Magnesium sulphate for foetal neuroprotection was administered when delivery was expected between 23 + 0 and 32 + 0 weeks. A new course of antibiotics was not routinely administered, except for cases of suspected clinical chorioamnionitis.

Patients who remained stable were discharged and followed on an outpatient regimen, which included a weekly clinical evaluation, including cervical length, MVP-AF monitoring, and CRP and WBC determinations, until delivery.

If severe and prolonged oligohydramnios (MVP-AF < 1 cm for a week after PPROM) was present before 22 + 6 weeks, extensive medical counselling, including risks and benefits of expectant management, selective or entire termination of pregnancy, was performed according to local legislation, which permits foetal or pregnancy termination in cases of severe and prolonged oligohydramnios until 22 + 6 weeks.

Induction of labour was indicated after 34 + 0 weeks or on diagnosis of non-reassuring foetal status, clinical chorioamnionitis, placental abruption, or cord prolapse, whichever occurred first.

### 2.5. Statistical Analysis

Continuous variables were presented as median with interquartile range (25th; 75th percentile). Categorical variables were presented as numbers with percentages (%). The Wilcoxon test was used to compare continuous variables, whereas the Chi-square or Fisher exact test was used to compare categorical variables. Univariate logistic regression was performed to evaluate independent clinical factors for predicting perinatal mortality or reaching periviable delivery. Differences were considered statistically significant when *p* < 0.05. Statistical analysis was performed using Stata for Mac statistical package (version 15.1 software, StataCorp.,College Station, TX, USA).

## 3. Results

Over the study period, 45 patients with twin pregnancies diagnosed with previable PPROM were admitted to our centre. Of these, 7/45 (15.6%) patients delivered within the following 24 h after PPROM. The maternal and gestational characteristics of the remaining 38 patients are shown in Table 1. Dichorionic pregnancies presented a higher incidence of ART. There was a higher rate of severe oligohydramnios (defined as MVP-AF < 1 cm) of the affected foetus in the dichorionic pregnancy group.

Overall, the median gestational age at PPROM was 18.6 (16.0–22.0) weeks. PPROM of the presenting foetus was reported in 32/38 (84.2%) of the cases, while PPROM of the non-presenting foetus was present in 4/38 (10.5%) of cases, and in 2/38 (5.3%) of cases, PPROM was observed in both amniotic sacs at admission.

**Figure 1:**
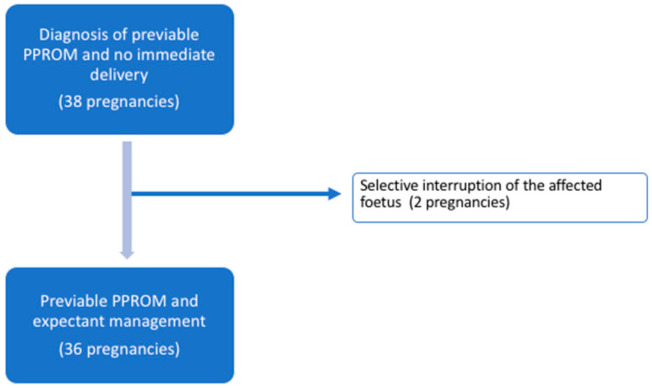
Flowchart of study population.

Two of the 38 (5.3%) patients opted for selective termination of the affected twin because of severe and prolonged oligohydramnios. These cases were excluded from the final neonatal analysis, as the outcomes might be substantially different from those with conservative management, and two cases do not allow an in-depth analysis. Therefore, 36 twin pregnancies opted for expectant management (Figure 1).

In the 36 on-going pregnancies, median gestational ages at delivery and latency from PPROM to delivery were 25.4 (22.1–28.6) and 5.4 (2.6–9.6) weeks, respectively (Table 2). A total of 25 of the 36 (69.4%) patients delivered after 23 + 0 weeks of pregnancy. A total of 16 out of 36 patients (44.4%) required tocolytic administration during pregnancy due to uterine contractions, after excluding any infectious suspicion of chorioamnionitis. 

Amniocentesis, to rule out intra-amniotic infection (IAI), was performed during pregnancy in 8/36 (22.2%) patients: 3 cases due to signs of clinical chorioamnionitis and 5 cases due to preterm labour and suspected infection in blood test results. In two of the latter cases, *Streptococcus agalactiae* and *Ureaplasma* spp. were isolated. In the first case, induction of labour, under an antibiotic regimen, was recommended due to the virulence of the microorganism, at 27.2 weeks, 8 weeks after PPROM, while in the second case, spontaneous delivery occurred at 21.6 weeks, 6 weeks after the diagnosis of PPROM. 

There were four cases of delayed-interval delivery, with a latency to delivery between twins of 9.5 (8;14) days. All of these cases delivered the first twin after periviability (at 23 + 0, 24 + 5, 26 + 3, and 28 + 6 weeks of pregnancy), but both twins survived in only one of four cases. Complications related to previable PPROM and maternal and neonatal morbidity are described in Table 2. 

Among the 36 ongoing pregnancies, in 21/36 (58%) cases, there was at least 1 survivor, and in 14/36 (38.9%) cases, both foetuses survived. Therefore, the overall survival rate was 35/72 (48.6%).

**Figure 2:**
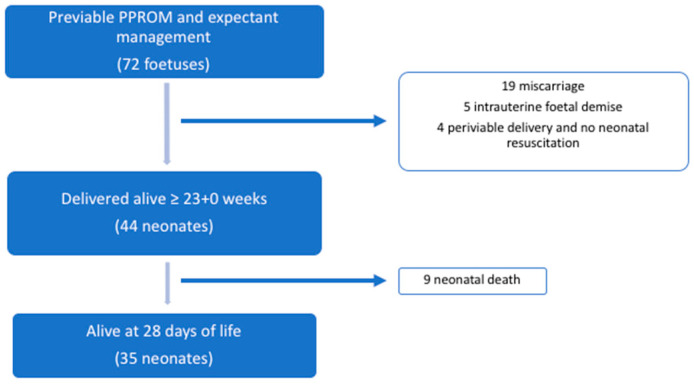
Flowchart of perinatal survival.

Periviability, considered as delivery from 23 + 0 weeks above, was achieved in 48/72 (66.7%) foetuses. Of these foetuses, 6/72 (8.3%) were delivered between 23 + 0 and 23 + 6 weeks. Two received corticosteroids for lung maturation and underwent active neonatal resuscitation at delivery due to a parental decision after extensive medical counselling. The remaining four foetuses were born without signs of life or received neonatal palliative care (Figure 2). Therefore, in the 44 foetuses born after 23 + 0 weeks and managed actively, neonatal survival increased up to 35/44 (79.5%), with at least 1 survivor in 21/25 (84%) and 2 survivors in 14/25 (56%) of the pregnancies.

Monochorionicity, use of ART, previous invasive procedure or foetoscopy, gestational age at PPROM and delivery, severe oligohydramnios, cervical length, IUFD of one twin, CRP, and leucocytosis (defined as WBC count > 11.0 × 10^9^/L) at admission were evaluated as independent factors for perinatal mortality or achieving periviability. Table 3 and Table 4 show that no factors, except for gestational age at delivery, were statistically significant predictors of perinatal mortality.

Taking into account that 16/38 (42.1%) of all previable PPROM cases were foetoscopy-related, gestational and perinatal outcomes between the two populations were compared. Except for the presence of severe oligohydramnios being more frequent in the non-foetoscopy group, no significant differences in terms of perinatal outcomes were observed between the two groups (Appendix A: Table A1).

## 4. Discussion

This study confirms that previable PPROM in twin pregnancies is associated with overall poor neonatal survival, mainly due to preterm delivery. However, our results show that when periviability is achieved, neonatal survival can significantly improve to up to 80%. Moreover, if delivery does not occur within the first 24 h of PPROM, the probability of achieving periviability, and thereby improving neonatal survival, is nearly 70%. 

Our findings are in line with most recent series. In 2016, Wagner et al. reported the perinatal outcomes of a series of 27 dichorionic twins with previable PPROM and expectant management. They described an overall survival rate (52%) and miscarriage rate (37%) quite similar to ours [3]. Wong et al. published a series of 23 dichorionic pregnancies complicated with PPROM before 26 weeks, recruited between 2002 and 2013, also with poor overall survival (43%) [4]. 

The gestational age at PPROM in our study was similar to that described by Wagner et al. but lower than Wong et al., which included PPROM until 26 weeks. However, our population presented a higher latency to delivery compared to these series. This could be explained, in part, by the fact that both authors included cases delivering within the first 24 h, and Wong et al. excluded delayed-interval delivery between twins. Both authors excluded monochorionic pregnancies and those with previous invasive procedures. Despite the differences in inclusion criteria among series, the perinatal results are quite similar, reinforcing our results independently of chorionicity or the performance of an invasive procedure. 

The percentage of pregnancies that reached viability in the present study was similar to previous series and was associated with an 80% probability of neonatal survival, in line with the results of Wagner et al. (90%) and much higher than those of Wong et al. (59%), probably due to improvement in neonatal care management in recent series. These differences may also be explained by changes in the management of preterm labour, which includes amniocentesis to rule out intra-amniotic infection and the wide use of tocolytics in our study population. Despite this, we acknowledge we did not perform a systematic amniocentesis to rule out infection in all our cases; in our casuistry, infection was confirmed in only 2/8 suspected cases, with infection being ruled out in the remaining 6 cases. Therefore, the indication for delivery was safely postponed, which could have potentially prolonged latency to delivery.

Patients diagnosed with previable PPROM, in whom there is no medical indication for immediate delivery or elective termination of pregnancy, should receive medical counselling regarding the risks and benefits of expectant management [5]. In singleton pregnancies, there are quite well-known prognostic factors for predicting perinatal mortality in previable PPROM, with latency and gestational age to delivery and severe and prolonged oligohydramnios among the strongest factors [6,9,10]. However, in twin pregnancies, due to the rare condition of previable PPROM, inconsistent data are available in terms of the prediction of latency to delivery and perinatal outcomes. We were not able to demonstrate predictive factors for perinatal mortality apart from gestational age at delivery. Similar to singleton pregnancies [6,10], a longer latency from PPROM to delivery and a higher gestational age at delivery are considered strong determinants for predicting perinatal survival in twin pregnancies. In our study, reaching periviability increased the likelihood of survival from 50 to 80% and the probability of taking home at least one child from 58 to 84%. To date, this is probably the only consistent and compelling information in prenatal counselling of these pregnancies. Although in our series, 70% of patients reached periviability, no prognostic factors could be identified as individual predictors for achieving this gestational age. Further research including a larger cohort is required to prospectively evaluate individual prognostic factors for perinatal mortality in twins complicated with previable PPROM. 

Although gestational age at PPROM has been suggested as a potential contributor to latency to delivery and perinatal survival, in either twin or singleton pregnancies [14,15,16], in the present study, gestational age at PPROM did not demonstrate to be an independent factor for perinatal mortality or delivery at ≥23 + 0 weeks.

Considering that the aetiology of previable PPROM in twin pregnancy may differ from that of a singleton pregnancy, chorionicity and the need for invasive procedures could be suggested as potential prognostic factors. Our study included a significant proportion of foetoscopy-related PPROM, which represents 76% of the aetiology of previable PPROM in the monochorionic pregnancy group, whereas, in the dichorionic group, 29% of the patients had undergone a previous invasive procedure. Nevertheless, neither chorionicity nor a history of a previous invasive procedure was an independent factor for perinatal mortality or delivery after 23 weeks. Similar to Bartin et al. [17], previable PPROM in foetoscopy cases occurred at an earlier gestational age at laser therapy (16.8 (16.2–17.7)). Blood test alterations, suggesting an infectious aetiology, were not useful as mortality or latency predictors, thereby suggesting a low incidence of IAI as a cause of previable PPROM in twins. Along the same line, the proportion of chorioamnionitis in our twin pregnancies was 22%, which is lower than in the singleton series (35%) [5,6].

Differences in the proportion of severe oligohydramnios were noted between pregnancy chorionicity, which could be explained by the mechanism of PPROM, because in monochorionic pregnancies, there was a higher proportion of iatrogenic PPROM than in dichorionic pregnancies, suggesting that in procedure-related PPROM amniotic fluid could be more preserved. On the other hand, and in contrast to what is known in singleton pregnancies, amniotic fluid volume did not seem to play such an important role in perinatal mortality. The rate of pulmonary hypoplasia in the present study seems to have been at the lower limit compared to what is reported in series comprising singleton pregnancies [5], but similar to our singleton data [6].

The main strength of this study is that, to our knowledge, this study evaluates one of the largest cohorts of twin pregnancies complicated with PPROM before viability and managed expectantly. Indeed, it is the first to assess risk factors for predicting perinatal mortality exclusively in this population. In addition, this is the first study to use the additional information provided by amniocentesis in cases of suspected infection. This could provide a selection of patients who would benefit from prolonging pregnancy by ruling out IAI, which could lead to severe infectious complications such as chorioamnionitis or maternal sepsis. Although amniocentesis is not routinely performed in twins due to the lower incidence of IAI [18], this might be an excellent tool in cases in which infection is suspected.

We also have to acknowledge some limitations of this study, one of which is its retrospective nature. Moreover, the sample size was limited to strongly demonstrate significant predictive factors for latency to delivery or mortality. However, considering that early PPROM is a rare event, especially in twins, studies including a larger number of individuals may need time for patient recruitment, and therefore, this study might be of help for assessment in the clinical setting. In addition, because our centre is a tertiary referral hospital, our cohort included a relevant number of monochorionic pregnancies complicated with foetoscopy-related PPROM. However, although this could be considered a potential selection bias and make our results less generalizable, our data show that chorionicity and foetoscopy within the monochorionic group do not seem to be crucial prognostic factors. Moreover, no significant differences in terms of perinatal outcomes were observed between the two populations. Finally, the study design made not possible a proper long-term neonatal follow-up assessment.

## 5. Conclusions

In conclusion, our retrospective data reinforce the fact that the overall survival rate in twin pregnancies complicated with previable PPROM is around 50%, similar to that of singleton pregnancies. However, once viability is reached, survival increases up to 80%. No prognostic factors, apart from achieving periviability, demonstrated significance as individual predictors for perinatal mortality.

## Figures and Tables

**Table 1:** Maternal and gestational characteristics.

Variables	Twin Pregnancies (N = 38)	Dichorionic (N = 17)	Monochorionic (N = 21)	*p*-Value
Maternal age (years)	32.4 (30.1- 37.2)	34.4 (30.7–37.9)	31.1 (29.2–36.9)	0.138
Ethnicity				
- Caucasian - Maghreb - Hispanic - Asian	32/38 (84.2) 3/38 (7.9) 2/38 (5.3) 1/38 (2.6)	14/17 (82.3) 1/17 (5.9) 1/17 (5.9) 1/17 (5.9)	18/21 (85.7) 2/21 (9.5) 1/21 (4.8) 0/21 (0)	0.882
Body mass index	24.7 (20.3–29.7)	27.9 (22.5–35.7)	22.6 (18.4–25.5)	0.083
ART	13/38 (34.2)	11/17 (64.7)	2/21 (9.5)	0.001
Nulliparity	25/38 (65.8)	13/17 (76.5)	12/21 (57.1)	0.307
SPTB * risk factors	4/38 (10.5)	1/17 (5.9)	3/21 (14.3)	0.613
Previous invasive procedure † GA at procedure	7/38 (18.4) 13.6 (12.0–20.0)	5/17 (29.4) 13.6 (12–16)	2/21 (9.5) 16.6 (13.3–20)	0.207 0.699
Previous foetoscopy GA at foetoscopy			16/21 (76.2) 16.8 (16.2–17.7)	NA NA
GA at PPROM	18.6 (16.0–22.0)	16.6 (15–20.9)	19.3 (16.7–22.3)	0.146
MVP-AF (mm) at PPROM	10 (0–20.5)	0 (0–15)	17 (10–26)	0.012
MVP-AF (mm) 1 week after PPROM	10 (0–19)	0 (0–10)	16.5 (10–25)	0.014
Cervical length (mm)	33.5 (25–40)	31 (25–40)	37 (23–41)	0.520

Continuous variables were compared using non-parametric Wilcoxon test and presented as median (25th; 75th percentile). Categorical variables were compared using Chi-squared or Fisher exact tests and presented as numbers (percentages). *p*-value between dichorionic and monochorionic groups. Abbreviations: ART—assisted reproductive technology, SPTB—spontaneous preterm birth, GA—gestational age, PPROM—preterm prelabour rupture of membranes, MVP-AF—maximum vertical pocket of amniotic fluid. * Factors for SPTB: smoking, previous SPTB, uterine malformation, cone biopsy. † Invasive procedure: chorionic villous sampling, amniocentesis, cervical cerclage.

**Table 2:** Perinatal and maternal outcomes of previable PPROM in twins.

Variables	Twin Pregnancies (N = 36)
Gestational age at delivery	25.4 (22.1–28.6)
Latency to delivery (weeks)	5.4 (2.6–9.6)
Chorioamnionitis Placental abruption Cord prolapse	8/36 (22.2%) 1/36 (2.8%) 2/36 (5.6%)
Maternal morbidity *	6/36 (16.7%)
	Foetuses (N = 72)
Intrauterine foetal demise Miscarriage (<23 + 0 weeks)	5/72 (6.9%) 19/72 (26.4%)
Overall perinatal survival Neonatal survival if delivery ≥23 + 0 weeks †	35/72 (48.6%) 35/44 (79.5%)
Birthweight (grams) † Neonatal death † Composite neonatal morbidity ǂ	988.5 (800–1260) 9/44 (20.5%) 22/44 (50%)
Pulmonary hypoplasia	4/44 (9.1%)

Data are presented as median (25th; 75th percentile) or number (percentage). * Maternal morbidity: severe postpartum haemorrhage, hysterectomy, infectious complications, maternal intensive care unit admission. † Includes only foetuses with active neonatal management. ǂ Composite neonatal morbidity: bronchopulmonary dysplasia, early sepsis, III–IV stage intraventricular haemorrhage, necrotizing enterocolitis, and neonatal death.

**Table 3:** Univariate analysis of factors associated with perinatal mortality.

Variables	OR (95% Confidence Interval)	*p*-Value
Monochorionicity	1.65 (0.41–6.68)	0.481
Assisted reproductive technology	1.30 (0.32–5.33)	0.719
Previous invasive procedure * Foetoscopy	2.33 (0.36–15.05) 1.71 (0.40–7.27)	0.373 0.467
Gestational age at PPROM	1.04 (0.83–1.30)	0.716
MVP-AF < 1 cm at PPROM diagnosis MVP-AF < 1 cm after 1-week of PPROM	1.33 (0.28–6.28) 1.25 (0.21–7.62)	0.716 0.809
Cervical length < 25 mm at PPROM	0.54 (0.09–3.14)	0.498
Intrauterine foetal demise of one twin	1.00 (0.12–8.13)	>0.99
C-reactive protein levels at PPROM Leucocytosis † at PPROM	1.63 (0.74–3.59) 1.50 (0.26–8.82)	0.222 0.654
Gestational age at delivery	0.22 (0.06–0.75)	0.016

Data are presented in odds ratio (OR) (95% confidence interval). Abbreviations: PPROMs—preterm prelabour rupture of membranes, MVP-AF—maximum vertical pocket of amniotic fluid. * Excludes foetoscopy. † Defined as white blood cell count > 11.0 × 10^9^/L.

**Table 4:** Univariate analysis of factors associated with delivery ≥23 + 0 weeks.

**Variables**	**OR (95% Confidence Interval)**	***p*** **-Value**
Monochorionicity	0.30 (0.06–1.38)	0.121
ART	3.54 (0.63–19.82)	0.151
Previous invasive procedure * Foetoscopy	0.51 (0.09–2.79) 0.39 (0.09–1.68)	0.436 0.207
GA at PPROM	1.23 (0.97–1.56)	0.094
MVP-AF < 1 cm at PPROM diagnosis MVP-AF < 1 cm after 1-week of PPROM	1.67 (0.35–8.04) 3.21 (0.47–21.80)	0.525 0.232
Cervical length < 25 mm at PPROM	0.39 (0.07–2.04)	0.265
Intrauterine foetal demise of one twin	0.23 (0.03–1.65)	0.144
C-reactive protein levels at PPROM Leucocytosis † at PPROM	1.11 (0.58–2.14) 1.67 (0.29–9.44)	0.748 0.564

Data are presented in odds ratio (OR) (95% confidence interval). Abbreviations: PPROMs—preterm prelabour rupture of membranes, MVP-AF—maximum vertical pocket of amniotic fluid. * Excludes foetoscopy. † Defined as white blood cell count > 11.0 × 10^9^/L.

## Data Availability

The data presented in this study are available on request from the corresponding author.

## Appendix A

**Table A1:** Gestational and perinatal outcomes in foetoscopy versus no foetoscopy groups.

**Variables**	**No Foetoscopy (N = 22)**	**Foetoscopy (N = 16)**	***p*** **-Value**
Gestational age at PPROM (weeks)	17.9 (15.0–21.6)	18.8 (16.8–22.6)	0.147
Previous invasive procedure	6/22 (27.3)	1/16 (6.3)	0.203
MVP-AF (mm) at PPROM MVP-AF (mm) 1 week after PPROM	0 (0–13.5) 6 (0–15)	19.5 (13–26) 15.5 (0–25)	0.007 0.218
Cervical length (mm)	30.5 (21.5–37)	37.5 (32–44.5)	0.054
Selective termination	0 (0)	2/16 (12.5)	0.171
Gestational age at delivery (weeks)	26.5 (23–28.7)	23.9 (21–28.6)	0.417
Latency to delivery (weeks)	6.5 (3.6–12)	3.1 (2–8.6)	0.127
PPROM related complications *	8/22 (36.4)	3/14 (21.4)	0.467
Intrauterine foetal demise Miscarriage (<23 + 0 weeks)	3/44 (6.8) 9/44 (20.5)	2/28 (7.1) 10/28 (35.7)	>0.99 0.152
Overall perinatal survival Neonatal survival if delivery ≥23 + 0 weeks †	24/44 (54.6) 24/30 (80)	11/28 (39.3) 11/14 (78.6)	0.207 >0.99
Composite neonatal morbidity ǂ Pulmonary hypoplasia	15/30 (50) 4/30 (13.3)	7/14 (50) 0 (0)	>0.99 0.290

Continuous variables were compared using non-parametric Wilcoxon test and presented as median (25th; 75th percentile). Categorical variables were compared using Chi-squared or Fisher exact tests and presented as number (percentage). *p*-value between dichorionic and monochorionic groups. Abbreviations: PPROMs—preterm prelabour rupture of membranes, MVP-AF—maximum vertical pocket of amniotic fluid. * PPROM-related complications: chorioamnionitis, placental abruption, cord prolapse. † Includes only foetuses with active neonatal management. ǂ Composite neonatal morbidity: bronchopulmonary dysplasia, early sepsis, III–IV stage intraventricular haemorrhage, necrotizing enterocolitis, and neonatal death.
